# Supplementary material for: Influence of Te-Doping on Catalyst-Free VS InAs Nanowires
Source: Nanoscale Res Lett. 2019 May 28;14:179. doi: 10.1186/s11671-019-3004-0 (PMC6538743; doi:10.1186/s11671-019-3004-0)
Supplement: Supplementary file 1 — Figure S1. Schematic illustration of the NW device processing: (a) resist coating, (b) e-beam writing, (c) development, (d) metallization, (e) lift-off process, and (f) the resulting contacted nanowire. The inset in f) shows a SEM topview of a metal contacted InAs NW. Figure S2. SEM side images of the doped and undoped InAs nanowires. a) growth series A and b) growth series B. The GaTe-cell temperature is indicated for each image at the top left. (DOCX 1610 kb) [file 11671_2019_3004_MOESM1_ESM.docx]

**Supplementary material: Inﬂuence of Te-doping on catalyst-free VS InAs nanowires**

Nicholas A. Güsken^1,3^, Torsten Rieger^1,3^, Gregor Mussler^1,3^, Mihail Ion Lepsa^2,3,*^ and Detlev Grützmacher1^,2,3^

*Correspondence: m.lepsa@fz-juelich.de

^1^Peter Grünberg Institute (PGI-9), Forschungszentrum Jülich GmbH, 52425 Jülich, Germany.

^2^Peter Grünberg Institute (PGI-10), Forschungszentrum Jülich GmbH, 52425 Jülich, Germany.

^3^JARA-Fundamentals of Future Information Technology (JARA-FIT), Jülich-Aachen Research Alliance, Germany.


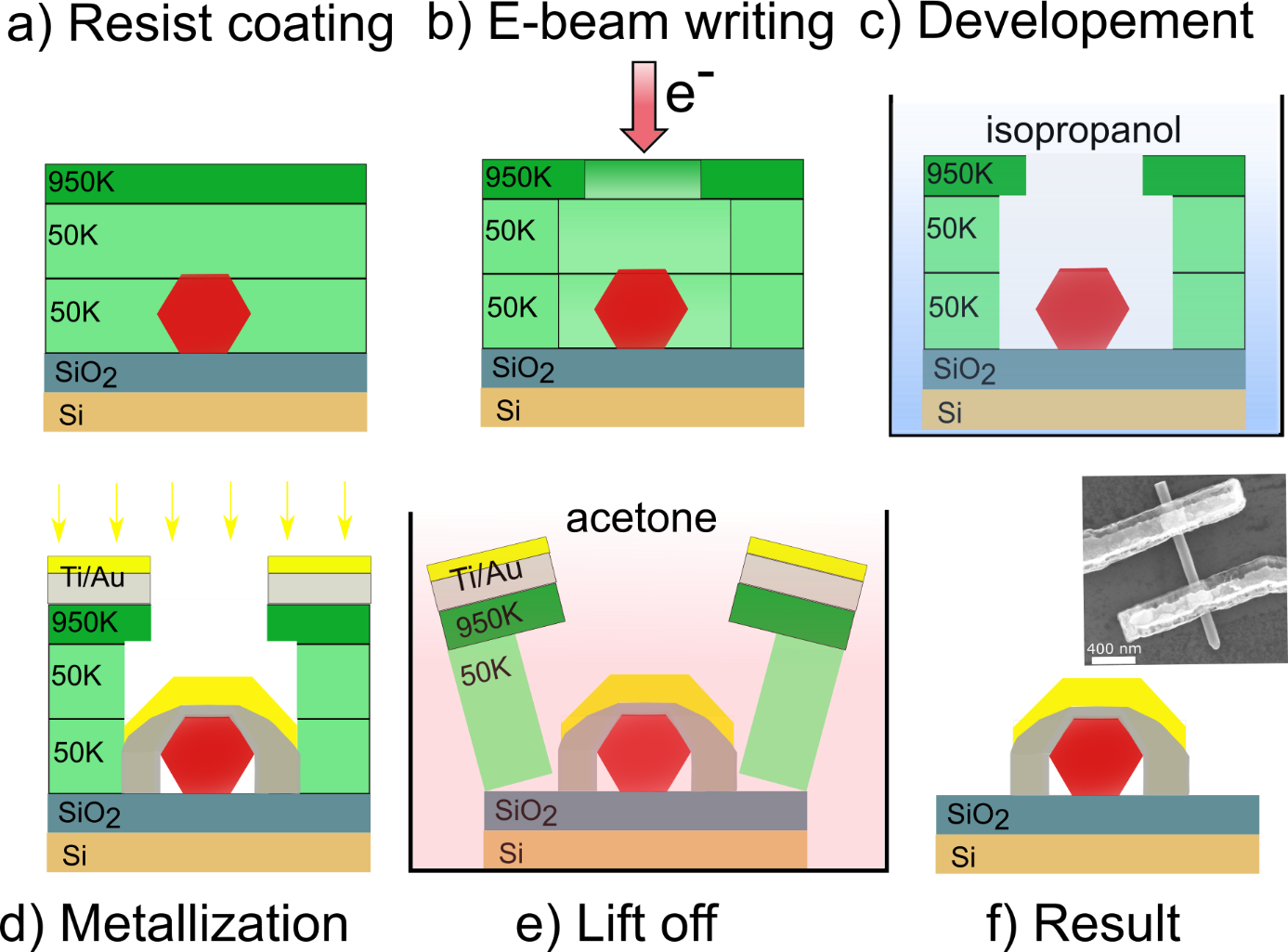


Figure S1: Schematic illustration of the NW device processing: (a) resist coating, (b) e-beam writing, (c) development, (d) metallization, (e) lift-off process, and (f) the resulting contacted nanowire. The inset in f) shows a SEM topview of a metal contacted InAs NW.


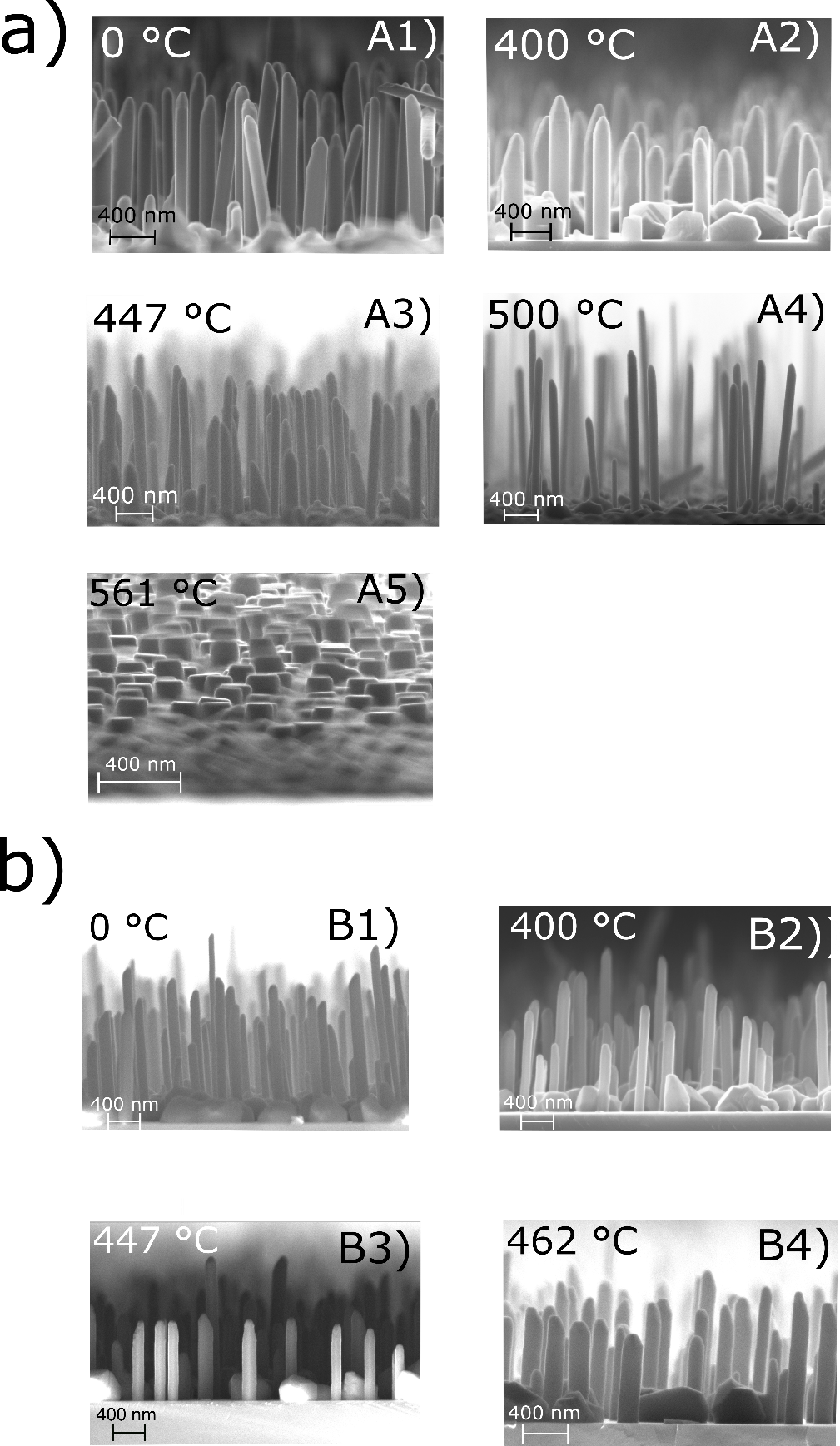


Figure S2: SEM side images of the doped and undoped InAs nanowires. a) growth series A and b) growth series B. The GaTe-cell temperature is indicated for each image at the top left.
